# Ethnic Differences in the Food Intake Patterns and Its Associated Factors of Adolescents in Kelantan, Malaysia

**DOI:** 10.3390/nu8090551

**Published:** 2016-09-12

**Authors:** Nurul-Fadhilah Abdullah, Pey Sze Teo, Leng Huat Foo

**Affiliations:** 1 Programme of Nutrition, School of Health Sciences, Universiti Sains Malaysia, Health Campus, Kubang Kerian, Kelantan 16150, Malaysia; nfha_9802@yahoo.com (N.-F.A.); peysze1985@msn.com (P.S.T.); 2 Department of Health Science, Faculty of Sport Science and Coaching, Universiti Pendidikan Sultan Idris, Proton City, Tanjung Malim, Perak 35900, Malaysia; 3 School of Biosciences, Taylor’s University lakeside campus, Subang Jaya, Selangor 47500, Malaysia

**Keywords:** dietary patterns, food-frequency questionnaire, dietary, lifestyle practices, adolescents

## Abstract

Objective: The aim of the study was to identify the ethnic differences in dietary patterns and its association with socio-economic, dietary and lifestyle practices among adolescents in Kelantan, Malaysia. Methods: A population-based study of 454 adolescents aged 12 to 19 years was included. A validated food frequency questionnaire was used to assess dietary patterns and three dietary patterns were identified based on the principal component analysis method. Results: Malay adolescents had significantly higher scores for the Western-based food pattern and local-based food pattern, whereas Chinese adolescents showed higher scores for the healthy-based food pattern. Multivariate analyses show that age and physical activity (PA) levels were positively associated with healthy-based food pattern in Malay (All, *p* < 0.001), whereas higher consumption of eating-out from home (EatOut) (*p* = 0.014) and fast food (*p* = 0.041) were negatively associated. High weekly breakfast skipping (*p* < 0.001) and EatOut (*p* = 0.003) were positively associated with a Western-based pattern, whereas age (*p* < 0.001) and household income (*p* = 0.005) were negatively associated. Higher frequency of daily snacking (*p* = 0.013) was positively associated with local-based food pattern. For Chinese adolescents, age (*p* < 0.001), PA levels (*p* < 0.001) and maternal education level (*p* = 0.035) showed positive associations with the healthy-based pattern, whereas high EatOut (*p* = 0.001) and fast food intakes (*p* = 0.001) were negatively associated. Higher weekly consumption of EatOut (*p* = 0.007), fast food (*p* = 0.023) and carbonated beverages (*p* = 0.023), and daily snacking practice (*p* = 0.004) were positively associated with higher Western-based food pattern, whereas age (*p* = 0.004) was inversely associated. Conclusion: This study showed that there were significant differences in dietary patterns and its association factors between Malay and Chinese adolescents. More importantly, these findings suggest that unhealthy dietary and lifestyle practices could increase the risk of adherence to unhealthy Western-based food pattern that is high in fat, sugar and salt contents, and, consequently, increase the risk of developing obesity and metabolic-related disorders during these critical years of growth.

## 1. Introduction

Habitual unhealthy eating and inactive lifestyle practices during childhood and adolescence can increase the risk of obesity, cardiovascular diseases, type 2 diabetes and various types of cancers in young people and also in later adult life [1]. Moreover, unhealthy dietary practices acquired in childhood may persist into adulthood [2]. This suggests that unhealthy dietary and lifestyle practices during the years of growth have significant implications for the health of individuals throughout life. Therefore, establishing healthy and balanced dietary practices in children and adolescents will ultimately be an important public health strategy to promote optimal nutritional status as well as in diminishing the risks of non-communicable diseases.

Although the impact of single nutrients and/or individual foods on health and disease risks have been extensively studied, the usefulness of this approach has often been questioned for understanding, at the population level, the complex interactions between diet and chronic disease development. This is partly due to the fact that people do not eat individual nutrients, rather they consume meals containing a variety of foods with complex combinations of nutrients that could act synergistically to influence the risk of chronic diseases [3,4]. The complexities of various diets make it difficult to consider the role of individual foods or nutrients in isolation. Hence, examining and understanding the relationships between diet and health status and/or chronic disease risks in populations over a lifetime, should take into account the complex interactions between nutrients and/or different food groups [3], using statistical analyses such as the principal component analysis (PCA) to define food patterns [5]. This is a data-driven modelling method to identify the foods that are frequently consumed. All food items were aggregated into factor-based food groups based on the degree to which the amount consumed is correlated with one another, after taking into account the substantial variation in diet composition between individuals [3]. 

Food consumption patterns are also influenced by a multitude of factors such as socio-demographic status, dietary behaviours and lifestyle practices [6,7]. Few studies that have examined the association between all these factors on food patterns among children and adolescents and they tend to report that a healthy food pattern, characterised by high intake of fruit, vegetables, and dairy products, is positively associated with higher socio-economic status (SES) and educational status [6,7,8], and was inversely associated with unhealthy lifestyle behaviours such as smoking, excessive alcohol consumption and low physical activity [6,7,8,9]. Most of these studies were carried-out with Caucasian children and adolescents, with only a few dealing with Asian children [10,11]. Two studies of Asian adolescents only focused on SES [10] and the contribution of food patterns on nutritional profiles [11]. In contrast, there is limited evidence between dietary behaviours and food patterns in Asian children. Understanding interrelationships of these socio-economic, dietary and lifestyle-related factors on dietary food patterns in children and adolescents is important because eating habits developed during early life could be a significant influence on lifelong eating behaviours. Healthy eating habits will ultimately improve nutritional well-being and consequently will diminish the risk of obesity and other metabolic-related disorders later in life. Moreover, identification of these modifiable dietary and lifestyle factors in relation to dietary patterns among adolescents would also help in devising cost-effective healthy nutrition intervention programs, targeting high-risk adolescents with unhealthy eating practices during their critical years of growth. Thus, the aim of the present study was to identify food patterns in adolescents aged 12 to 19 years and also to examine whether socio-economic characteristics, dietary and lifestyle behavioural practice factors on these food patterns after taking into account other known potential confounding factors. A secondary objective was to compare between Malay and Chinese because of the differences in their habitual food patterns consumed by these major ethnic groups in Malaysia.

## 2. Materials and Methods

### 2.1. Participants and Setting

This present population-based study was conducted among apparently healthy adolescents of Malay and Chinese origins aged 12 to 19 years in the Kelantan, Malaysia. The sample size of these participants was determined based on the primary outcome of bone health assessments [12]. Participants were recruited based on several community approaches namely, through advertisements, school and community announcements, and peer-to-peer referral in community areas. Eligible participants were selected if they were healthy and physically active, and having no clinical signs of bone-related disorder that could potentially prevent them from being physically active. Two girls were excluded from the final analysis due to missing data. In total, 454 adolescents comprising 236 Malay and 218 Chinese adolescents were included. 

### 2.2. Ethical Approval

The present study was approved by the Research Human Ethics Committee of Universiti Sains Malaysia (USM). Written informed consent was obtained from both participants and parents or guardians prior to the study.

### 2.3. Assessments of General Characteristics, Socio-Economic Status and Dietary Behaviours 

Details of the methodology protocols of this study have been published elsewhere [13,14]. In brief, a pre-piloted self-administered questionnaire was used to assess general characteristics, socio-economic and demographical characteristics, and dietary behaviours of the participants. Socio-economic status such as the parental occupation and educational status, and household income were collected. Dietary behaviour such as daily breakfast consumption, snacking practices, eating-out from home practice, consumption of carbonated and sweetened beverages, and use of nutritional supplements was also assessed. Detailed information on the frequency, types and amount taken were also gathered for these dietary behaviours.

### 2.4. Assessments of Lifestyle Physical Activity (PA) and Screen-Based Sedentary (SSR) Practices 

A validated self-administered computer-based-PA questionnaire (cPAQ) was used to assess the type, frequency and intensity of PA practiced by participants in the preceding year. Details of the validity and reproducibility of this cPAQ have been described elsewhere [15]. In brief, the cPAQ was used to collect frequency (h/day) of school-based, leisure-based and household-based activities. Information on participation in organized sports either in school or in leisure time was also gathered. A screen-based sedentary practice was estimated by total daily duration spent on screen-based activities. Participants were asked for detailed information on the time they spent each week in viewing television, playing video games and using computers and/or the Internet, both on local school days and weekend days.

### 2.5. Dietary Intake Assessment 

A validated semi-qualitative past one-year food frequency questionnaire (FFQ) was used to assess dietary intake [16]. In brief, the FFQ of 124 food items that were categorised into 15 food groups namely, rice and noodle dishes, cereal and wheat-based food, meat and poultry, seafood and shellfish, milk and dairy products, eggs, nuts and legumes, tuber, vegetables, fruits, local deserts “*kuih-muih*”, fast food, snack foods, spread and sauce, and beverages. Participants were asked to recall the foods consumed over the past one-year, using a comprehensive range of food photos and common household measures, to help quantify food items, with the assistance of trained interviewers. Detailed information on frequency and portion size usually served for each food item was obtained. Completed FFQs were then re-checked by a trained nutritionist for completeness and accuracy.

### 2.6. Statistical Analysis

The food patterns analysis approach has been widely used in epidemiological population-based studies to investigate the role of food consumption pattern on health and/or disease outcome risks [3]. In the present study, food patterns were obtained using the principal component analysis (PCA), which was based on daily intake of each food item collected from the FFQ. Several food items were categorized into a new food groups, especially those food items that have similarity or almost similar in terms of the food characteristic and/or function in order to reduce the complexity of the data and also because there is recommendation that for each variable entering factor analysis, the investigators must have 10 participants [17]. Therefore, for the 454 participants, the 44 new food groups or food items (Table 1) were included in the PCA to identify food patterns.

Exploratory factor analysis approach was carried-out to help to summarise and refine large datasets containing several variables, simultaneously into a small number of orthogonal variables to be then labelled as a “pattern”, in which this analysis approach showed to be considerably valid and highly correlated as compared to absolute nutrient levels calculated from the FFQ [18]. The reliability of factor analysis was assessed through the Kaiser–Meyer–Olkin test (KMO) measurement of sample adequacy and the Bartlett test of sphericity, whereby the values of the KMO test of >0.6 and the Bartlett’s test of sphericity of <0.05 were used to assess sampling adequacy and inter-correlations of variables [19], followed by exploratory factor analysis. This was applied to 44 food groups to determine the principle factors/components of the maximum fraction of variance explained by each food group. A graphical method of the Scree plot was then used to determine the number of factors with an eigenvalue of >1.25 and varimax rotation was applied to review the correlations between variables and factors. Food patterns were labelled according to the food groups or food items that had the greatest influence on the respective factor loadings. Foods with a loading greater than or equal to 0.30 on a factor were used to describe the dietary patterns, as suggested by most studies on dietary pattern analyses [6,20]. Factor scores were calculated by multiplying factor loadings with the corresponding standardised value of each food and summing across the food items. Each participant received an individual factor score to indicate the extent to which his/her diet conformed to the respective food patterns. A high factor score for a given pattern indicated a high intake of the foods constituting that food pattern, whereas a low score indicated low intake of those foods. These food patterns were then used as outcome variables to determine their association to socioeconomic status, dietary behaviours and lifestyle practices among these adolescents.

All of the variables were tested for normality by the Kolmogorov–Smirnov test and test of homogeneity of variance before any statistical comparisons were made. Descriptive statistics were reported as mean values ± SD for numerical variables and frequency and percentage for categorical variables, unless otherwise indicated. An independent *t*-test was used to assess the differences between ethnic groups for continuous variables, whereas the Chi-square test was used for categorical variables. Ethnicity-specific multiple linear regression analysis with a stepwise elimination method was used to determine the associations of socio-economic profile, dietary behaviours and lifestyle practices among these adolescents, after taking into account other known potential confounders such as gender and age. Where appropriate, interactions between dietary and lifestyle factors and food patterns were also tested. Data analyses were performed using the SPSS for windows version 20.0 (SPSS Inc., Chicago, IL, USA). A *p* value < 0.05 was considered to be significant.

## 3. Results

Table 2 shows the general characteristics of socio-economic status, dietary and lifestyle behaviours of the participants. The mean age of adolescents was 15.3 ± 1.90 years. Most of their parents had obtained educational levels up to secondary school. Ethnicity comparisons of the participants showed no significant difference in household income status between Malay and Chinese adolescents. In contrast, Chinese adolescents were from significantly smaller household sizes compared to their Malay counterparts (*p* < 0.001). There were significant differences reported in dietary practices such as weekly frequency of breakfast skipping (*p* < 0.001), eating-out from home (*p* = 0.03) and use of nutritional supplements (*p* < 0.001) between Malay and Chinese participants, in which almost half of the Malay adolescents (44%) were reported to have skipped breakfast at least once a week compared to only 21% of the Chinese adolescents. In contrast, Chinese adolescents had higher use of nutritional supplements per week than that of the Malays (36% vs. 15% per week). In terms of lifestyle practices, there was no difference found in physical activity levels between Malay and Chinese, but Chinese adolescents had significantly higher total screen-based SSR levels in a week (*p =* 0.02) compared to the Malay participants.

Three food patterns were identified by the PCA and labelled as Western-based, healthy-based and local-based food pattern, based on the factor loading extracted from each food item and/or food group (Table 3). The pattern labelled Western-based had higher loading factors for food items/groups that were characterised by high intakes of animal-based and processed foods, which were high in fat, salt and sugars. A local-based food pattern was identified based on higher factor loading of several common habitual foods consumed in these populations such as white rice, condensed sweetened milk, tea, seasoning fish sauce “*budu*”, banana fritter and white bread. On the other hand, food items with higher loading factors such as fruit, vegetables, dairy products, nut and cereal-based foods were identified and labelled as a healthy-based food pattern. These three food patterns explained approximately 26.2% of the total variations in total food intake with approximately 12.5%, 7.7% and 6.0% attributed to the Western-based, healthy-based and local-based food patterns, respectively.

Comparisons of these food patterns across ethnicity showed that Malay adolescents had significantly higher scores for the Western-based food pattern (*p* < 0.001) and local-based food pattern (*p* < 0.001) compared to the Chinese participants (Table 4). In contrast, Chinese adolescents showed higher scores for the healthy-based food pattern than the Malay adolescents (*p* = 0.039).

Table 5 shows the predictors of SES, dietary behaviours and lifestyle practice factors on food patterns in stepwise multiple regression analyses. There was some similarity in terms of dietary and lifestyle factors related to these food patterns among Malay and Chinese adolescents. For the healthy-based food pattern, age (*p* < 0.001) and total physical activity levels (*p* < 0.001) showed significantly and positively associated with healthy-based pattern in Malay adolescents, after full adjustments for socio-demographic and economic status, dietary and lifestyle factors. In contrast, weekly consumption of eating-out from home (*p =* 0.014) and fast food intakes (*p* = 0.041) were negatively associated with healthy-based food pattern. All of these factors explained about 15% of total variation in this healthy-based food pattern. Similar observations were found for Chinese adolescents, where age (*p* < 0.001), total physical activity levels (*p* < 0.001) and maternal educational status (*p* = 0.035) showed significantly and positively associations with this healthy-based food pattern, whereas weekly consumption of eating-out (*p* = 0.001) and fast food intake (*p =* 0.001) were negatively associated with healthy-based food patterns. All these factors explained about 29% of the total variance of this healthy-based food pattern model.

In the Western-based food patterns, higher frequency of weekly breakfast skipping (*p* < 0.001) and eating-out from home (*p =* 0.003) emerged as significantly and positively associated with this diet patterns, whereas age (*p* < 0.001) and household income levels (*p =* 0.005) were negatively associated with the Western-based food pattern in Malay adolescents. In addition, there were several dietary practices such as weekly frequency of fast food (*p* = 0.023), carbonated sweetened beverages (*p* = 0.023), eating-out from home (*p* = 0.007), and daily snacking practice (*p =* 0.004) that were found to be significantly associated with the Western-based food pattern in Chinese participants, whereas age (*p* = 0.004) was a significant negative association with this Western-based food pattern.

For the local-based food pattern that is often referred to as the “traditional-based” food pattern, the frequency of daily snacking intakes (*p* = 0.013) showed a significant positive association with local-based food pattern in Malay adolescents. In addition, higher frequency of eating-out from home (*p* = 0.003), fast food intakes (*p* = 0.021) and daily snacking intakes (*p* = 0.009) were shown to be positive associations with this local-based food pattern among Chinese adolescents, after full adjustments for age, gender, socio-economic status, dietary and lifestyle factors. A weekly use of nutritional supplement (*p* = 0.027) was a negatively and significantly associated with the local-based food pattern. In terms of general characteristics and socio-economic status, older adolescents showed greater adherence to the healthy-based food pattern, whereas being a younger participant was significantly associated with higher Western-based food pattern. Moreover, higher maternal educational status of the participants was significantly associated with closer adherence to a healthy-based food pattern in Chinese adolescents, whereas lower household income level was significantly associated with higher Western-based food pattern among Malay participants.

## 4. Discussion

The present study sought to identify dietary patterns of Malay and Chinese adolescents aged between 12 and 19 years in Malaysia, in which three major dietary food patterns have been identified by the PCA and labelled as healthy-based, Western-based and local-based food patterns. These food patterns showed some similarities with the food patterns analyses from other studies carried-out with children and adolescents from elsewhere [21,22,23]. The Western-based food pattern in our present study is comparable with several Western-based patterns reported in adolescents from Australia [21], Germany [22] and Korea [23], whereas the healthy-based food pattern identified in this study, characterised by high intakes of fruit, vegetables, dairy products, nut and cereal-based foods, shows some comparability in terms of the description of food groups present in their “healthy” patterns [21,22].

This is the first study that has examined the associations between socio-economic, dietary and lifestyle factors on food patterns in children and adolescents, particularly in adolescents from different ethnic backgrounds in Asia. Interestingly, there was a significant ethnic difference in these food patterns found among these adolescents. Numerous population-based studies have shown that marked differences across ethnic groups on the risks of non-communicable diseases [24,25], but very few studies so far have investigated the ethnic differences in dietary patterns among adolescents in Asian populations, including in Malaysia. Interestingly, the present findings show that there was a significant variation in these dietary patterns consumed by adolescents of these major ethnic groups, in which Chinese adolescents had higher intake of healthy-based food pattern than that of the Malay participants. In contrast, the Western-based and local-based dietary patterns were most common among Malay adolescents and may constitute a dietary pattern descriptive of local and traditional dietary habits. This ethnic difference in dietary patterns may possibly be a reflection of differences in socio-cultural in relation to food preferences.

Moreover, the present findings also indicate that habitual eating and lifestyle behaviour practices were significantly linked to food patterns among these adolescents. A healthy-based food pattern, positively associated with higher physical activity levels, and some dietary behaviours such as less frequent out-of-home eating and fast food intake, shows that both healthy eating and active lifestyle habits are important determinant factors of healthy-based food patterns in these adolescents. On the contrary, unhealthy dietary behavioural practices such as a high frequency of breakfast skipping, and high frequency of eating-out from home in a week, and intakes of fast foods, carbonated sweetened beverages and snacking in a day were significantly and positively associated with greater adherence to the “Western-based” food pattern among these adolescents. These findings are consistent with several studies of adolescents of comparable age from other countries [9,11,22,26]. In a study of Korean adolescents, those with high intakes of bread, noodles, cookies and pizza/hamburgers were more likely to consume more fast foods, fried foods and carbonated beverages as well as having a high frequency of eating-out from home [11]. Similarly, a study of Caucasian adolescents also found that high frequency of daily snacking and weekly eating of meals in fast food restaurants were positively asscoiated with higher soft drink intakes [26]. Higher consumption of snacking in a day was also reported to be positively associated with a higher “junk food” pattern, characterised by greater amounts of processed food and snack foods that were high in fat, salt and sugar by children at four years of age in the United Kingdom [27]. It was also found that daily snacking practice was associated with a high score of “traditional diet” pattern in these children [27]. This finding in that study is comparable with the present study where a local-based food pattern for usual or common types of food consumed by adolescents in Malaysia was positively associated with daily snacking practice, which is another novel finding of the present study.

Active lifestyle practices such as higher level of daily physical activity was significantly associated with greater adherence to the healthy-based food pattern in these adolescents. This finding is comparable to results from several other studies conducted among Caucasian children and adolescents [22,28,29]. Higher participation in physical activity levels had significant and positive associations with higher consumption of a Mediterranean diet pattern among Balearic Islands adolescents [28]. Similarly, in a study of Dutch adolescents aged 12 to 16 years, a positive association was reported between physical activity levels and healthy eating pattern, after taking into account other dietary and lifestyle factors [29]. Another study with German adolescents found that high level of physical activity was significantly associated with greater adherence towards the healthy food pattern in boys, whereas sedentary practices such as long periods of television viewing was positively associated with greater prevalence of a Western-based food pattern in girls [22]. However, in Scottish children aged 5 to 17 years, there was no such consistent association was found between physical activity levels and food patterns assessed [7]. However, greater time viewing television was negatively associated with the healthy-based food patterns among both boys and girls, and a positive association was found only with a pattern of high intakes of “puddings” and unhealthy “snacks” in girls [7]. We were unable to find any significant association between screen-based sedentary practices, as assessed by total duration of daily screen-based activity, and any of the food patterns assessed. This may be due to less variability in duration of sedentary practices among the adolescents in the present study.

Older adolescents had higher adherence with the healthy-based food pattern, whereas younger participants were significantly associated with higher consumption of the Western-based food pattern. These findings are consistent with numerous other studies carried-out among European adolescents [8,30]. Aranceta and colleagues reported that a higher proportion of older adolescents had adequate intakes of fruit, while the “snacky” pattern was positively associated with adolescents of a younger age [8]. A similar observation was also reported with adolescents from the Balearik Islands where a greater adherence to the “Western” pattern was found in younger adolescents compared to their older peers [30]. However, Australian adolescents with a higher intake of healthy food was more likely seen at a younger age [31]. One possible explanation for our findings could be that older adolescents have greater autonomy over their food intake and progressively start to make their own choice of food [32]. It could possibly also reflects the fact that older adolescents are more concerned about their body image [33], which might also explained the reason for choosing healthy food found in our present study.

The relationships between food patterns and various socio-economic indicators among children and adolescents are mixed. Some studies [7,8,34], but not others [11,31], have indicated that children from lower socio-economic status may be at higher risk of unhealthy food practices. In the present study, Malay adolescents with lower household income were associated with greater adherence to the Western-based dietary pattern. This finding is in accordance with a study of adolescents in Australia where low socioeconomic status was positively associated with the Western-based food pattern [34]. In contrast, Chinese adolescents whose mothers had higher levels of education had significantly greater adherence to the healthy-based food patterns [7]. Apart from a positive association between high maternal educational status and healthy-based food pattern found in the present study, there are several other reports that adolescents whose mothers had lower educational levels had significantly higher intakes of salty, fat and sugary food [7,8,35]. For instance, Ambrosini and her-colleagues reported that lower maternal educational status emerged as an important independent predictor of an unhealthy diet pattern that was high in energy-dense, high fat, low fibre foods among adolescents followed-up for seven years [35]. All of these results underline the prominent role of mothers in shaping their children’s dietary food habits in early life. Another interesting finding was observed for the local-based food pattern among Malay adolescents, in which only snacking practice was significantly associated with this local-based food patterns with a total variance of 2% compared to their Chinese counterparts. Other dietary and lifestyle factors were not associated with this food pattern in Malay participants. This result is consistent with the fact that local-based food pattern is mainly derived from Malay culture. This showed that socio-culture may also play an important role in determining the food preference. Hence, large population studies warrant further investigations on how the socio-cultural factors affect food preferences and food choices that could consequently influence the nutritional well-being among adolescents from diverse ethnicity groups in Malaysia.

Some limitations should be acknowledged in this present study. Firstly, factor analysis, as a statistical technique, requires some arbitrary decisions and subjective interpretation of factors. However, dietary patterns identified using factor analysis has been shown to be a reliable dietary assessment tool [17,36]. Furthermore, this statistical technique was considered to be suitable for behavioural research because it manages to capture combinations of nutrients and foods [37]. Secondly, there are limitations in using a FFQ related to individual measurement errors. However, the FFQ remains one of the most practical methods for epidemiological dietary studies. Furthermore, comparing the results of PCA using FFQ and weighed dietary records have found the resulting patterns to be similar [18]. Thirdly, the total variance attributed to these three dietary patterns in the present study was about 26.2%; the low variance could possibly be suggested the potential existence of other patterns [38]. On the other hand, our findings are comparable with several previous epidemiologic studies on dietary patterns in adolescents of comparable age in other countries [30,31]. Lastly, because of the cross-sectional design of this study, causality effects for associations between dietary patterns and socio-demographic, dietary and lifestyle factors in these adolescents cannot be established. Nevertheless, there are some strengths of this study that also need to be highlighted. It investigated a range of habitual dietary and lifestyle practices such as daily breakfast consumption, snacking practices, eating-out from home practices, consumption of carbonated and sweetened beverages, the use of nutritional supplements, physical activity and SSR, and, to our knowledge, this is the first study that has conducted such a comprehensive investigation. In addition, a large sample size with a wide ages and ethnicity were strengths of this study.

## 5. Conclusions 

These findings suggest that unhealthy dietary behaviours and lifestyle practices by adolescents could increase the risk of perpetuating an unhealthy Western-style food pattern, characterised by high intake of animal-based and processed foods with a consequent increase in the risk of developing obesity and metabolic-related disorders. On the other hand, healthy dietary behaviours such as lower consumption of eating-out from home and fast food and more active lifestyle practices such as high levels of physical activity are important determinants of healthy-based food patterns in these adolescents. These findings indicate the importance that any intervention programs aiming to promote the consumption of healthy balanced diets in growing adolescents take into account the differences in dietary behaviours and lifestyle practices of adolescents across different ethnic groups when designing promotional strategies. Future research requires further investigation of the negative consequences of these food patterns on health outcomes among growing adolescents during these critical periods of growth.

## Figures and Tables

**Table 1:** Food group used in the principal component analysis (PCA).

Food Group	Items
White rice	
White bread	
Whole grains	Wholemeal bread, breakfast cereals, oat
Biscuits	Marie, cream crackers
Rice dishes	Fried rice, chicken rice, *dagang* rice, coconut rice, *kerabu* rice
Noodle dishes	*Mee, meehoon, kuehteaw*
*Roti canai* ^†^	
Chicken	
Red meat	Beef, lamb, pork
Internal organ	Beef, chicken
Fish	Whole, fillet
Seafood	Shrimp, cuttlefish, crab, cockles
Egg	
Salted egg	
Soy bean products	*Tauhu,* bean curb*,* soy milk
Nuts	Peanut, cashew nut
Processed meats	Nugget, sausage, burger
Pizza	
Coleslaw	
Green leafy vegetables	Green mustard, spinach, water spinach, kale
Cruciferous vegetables	Cabbage, cauliflower, broccoli
Other vegetables	Eggplant, tomato, cucumber
Legumes	Dhal gravy, gram soup, string bean, okra, mung bean
*Acar*	Mix of raw carrot, cucumber and pineapple in vinegar
Potatoes	French fries, mashed potato, fried, sweet potato fritter
Local tropical fruits	Papaya, banana, watermelon, honeydew, pineapple, guava, mango, jackfruit
Citrus fruit	Orange, apple, Asian pear, pear, grape
Seasonal fruits	Durian, mangosteen, rambutan, *duku, longan*
Dried fruit	Raisin, date
Sweet local dessert	*Akok*, *sri muka*, *kuih lapis*, doughnut, cake
Fried local dessert	*Cucur*, sardine roll, fried spring roll, *rempeyek*, curry puff, *cekodok*
Banana fritter	
Confectionery food	Ice-cream, milk chocolate
Condensed sweet milk	
Milk and dairy products	Fresh, full cream, low fat, flavoured, powder, yoghurt
Coffee	
Tea	
Chocolate malt drinks	
Carbonated and sweetened drinks	Carbonated soft drinks, cordial, fruit cordial, fruit squash, fruit syrup
Fish crackers	Ready-made cracker, *lekor* (local made fish cracker)
Salty snack food	Corn crackers, potato chips
Sweet bread spread	Fruits spread, coconut-egg jam *(kaya)*
Fat spread	Butter, margarine, peanut butter
Seasoning sauce	Soy sauce, *budu* (fermented anchovy with high content salt)

^†^ *Roti canai*—flatbread composed of dough containing copious amounts of fat (ghee-clarified butter), egg, flour and water.

**Table 2:** Socio-economic status, dietary behaviour and lifestyle practices of participant by ethnicity.

Characteristics	Malay (*n* = 236)	Chinese (*n* = 218)	*p* Value
	Mean ± SD/*n* (%)		
Age (years)	15.3 ± 1.9	15.3 ± 1.9	0.951
Gender, boys	104 (44.1)	100 (45.9)	0.699
**Socio-economic characteristics**			
Paternal education status			0.009
Primary education and below	25 (10.6)	34 (15.6)	
Secondary education	144 (61.0)	147 (67.4)	
Tertiary education	67 (28.4)	37 (17.0)	
Maternal education status			0.162
Primary education and below	23 (9.7)	26 (11.9)	
Secondary education	160 (67.8)	158 (72.5)	
Tertiary education	53 (22.5)	34 (15.6)	
Household size	6.8 ± 2.4	5.4 ± 1.3	0.000
Household income status			0.782
Low (<RM2300)	165 (69.9)	148 (67.9)	
Middle (RM2300–5599)	51 (21.6)	53 (24.3)	
High (>RM5600)	20 (8.5)	17 (7.8)	
**Dietary behaviours**			
Breakfast skipping (≤2 times/weeks) (Yes)	103 (43.6)	46 (21.1)	0.000
Fast food consumption (times/week)	1.1 ± 0.7	1.1 ± 0.9	0.522
Carbonated drink consumption (times/week)	3.7 ± 1.4	3.9 ± 1.6	0.248
Snacking practices (times/day)	2.1 ± 1.1	2.0 ± 1.0	0164
Eating-out from home practice (times/week)	2.8 ± 1.8	3.2 ± 2.2	0.030
Daily Nutritional supplement use (Yes)	35 (14.8)	79 (36.2)	0.000
**Lifestyle practices**			
Physical activity (PA) (h/day)	2.8 ± 1.7	3.0 ± 2.3	0.442
Small screen recreation (SSR) (h/day)	3.2 ± 1.9	3.7 ±1.8	0.021

**Table 3:** List of factors loading of food patterns.

Food Groups	Food Patterns		
	Western-Based	Healthy-Based	Local-Based
Noodle dishes	0.657		
Sweet local dessert	0.648		
Fried local dessert	0.623		
Rice dishes	0.566		
Confectionery food	0.565		
Chicken	0.536		
Red meat	0.520		
Fish	0.512		
Processed meats	0.509		
Salted egg	0.489		
Pizza	0.484		
*Roti canai*	0.471		
Seafood	0.471		
Fish crackers	0.458		
Salty snack	0.453		
Carbonated and sweetened beverages	0.403		
Internal organ	0.398		
Egg	0.395		
Biscuits	0.366		
Legumes		0.563	
Local tropical fruits		0.530	
Cruciferous vegetables		0.502	
Potatoes		0.491	
Green leafy vegetables		0.480	
Other vegetables		0.478	
*Acar*		0.449	
Soy bean products		0.447	
Milk and dairy products		0.440	
Dried fruits		0.411	
Citrus fruits		0.396	
Whole grains		0.354	
Seasonal fruit		0.327	
Nuts		0.314	
Chocolate malt drinks			0.532
Tea			0.529
Coffee			0.410
Condensed sweet milk			0.390
Banana fritter			0.373
Sweet bread spread			0.367
White bread			0.361
White rice			0.320
Fat spread			0.308
Seasoning fish sauce (*budu*)			0.246
Variance explained (%)	12.5	7.7	6.0

**Table 4:** Food patterns score of participant by ethnicity.

Food Patterns	Malay (*n* = 236)	Chinese (*n* = 218)	*p* Value
	Mean ± SD		
Healthy-based food pattern	−0.101± 0.957	0.094 ± 1.033	0.039
Western-based food pattern	0.224 ± 1.043	−0.239 ± 0.894	0.000
Local-based food pattern	0.399 ± 1.050	−0.427 ± 0.733	0.000

**Table 5:** Correlates of socio-economic characteristics, dietary behaviours and lifestyle practices on food pattern in adolescents by ethnicity ^1^.

	Regression Coefficient	Standard Error (SE)	Standard Coefficient (β)	*p* Value
**Healthy-based food pattern**				
**Malay adolescents**				
Age	0.141	0.033	0.262	0.000
Physical activity	0.142	0.036	0.239	0.000
Eating out from home practice	−0.088	0.036	−0.149	0.014
Fast food consumption	−0.166	0.081	−0.124	0.041
Constant	−2.044	0.551		0.000
Adjusted *R^2^* = 0.15				
**Chinese adolescents**				
Age	0.165	0.029	0.332	0.000
Eating out from home practice	−0.086	0.026	−0.194	0.001
Physical activity	0.100	0.024	0.243	0.000
Fast food consumption	−0.223	0.068	−0.198	0.001
Maternal educational level	0.242	0.114	0.127	0.035
Constant	−2.536	0.438		0.000
Adjusted *R^2^* = 0.29				
**Western-based food pattern**				
**Malay adolescents**				
Age	−0.136	0.033	−0.249	0.000
Breakfast skipping	0.476	0.129	0.227	0.000
Eating out from home practice	0.109	0.036	0.183	0.003
Household income	−0.078	0.027	−0.176	0.005
Constant	2.324	0.569		0.000
Adjusted *R^2^* = 0.14				
**Chinese adolescents**				
Age	−0.084	0.029	−0.182	0.004
Fast food consumption	0.156	0.068	0.149	0.023
Snacking practices	0.157	0.055	0.181	0.004
Eating out from home practice	0.072	0.026	0.174	0.007
Carbonated drink consumption	0.080	0.035	0.144	0.023
Constant	0.032	0.497		0.950
Adjusted *R^2^* = 0.18				
**Local-based food pattern**				
**Malay adolescents**				
Snacking practices	0.158	0.063	0.162	0.013
Constant	0.065	0.149		0.662
Adjusted *R*^2^ = 0.02				
**Chinese adolescents**				
Eating out from home practice	0.067	0.022	0.196	0.003
Snacking practices	0.254	0.096	0.173	0.009
Fast food consumption	0.133	0.057	0.155	0.021
Dietary supplement use	−0.216	0.097	−0.142	0.027
Constant	−0.569	0.121		0.000
Adjusted *R^2^* = 0.12				

^1^ Adjusting for gender (0 = boy; 1 = girl), age (years), maternal and parental education status, household size, household income, breakfast skipped (0 = not skippers; 1 = skippers), snacking (times/day), eating out (times/week), fast food consumption (times/week), soft drink consumption (times/week), dietary supplement use (0 = not use; 1 = use) and daily physical activity levels (h/day).
